# The Relationship between Plasma Erythropoietin Levels and Symptoms of Attention Deficit Hyperactivity Disorder

**DOI:** 10.1192/j.eurpsy.2023.629

**Published:** 2023-07-19

**Authors:** Y.-J. Kwon

**Affiliations:** Psychiatry, Soonchunhyang University Cheonan Hospital, Cheonan, Korea, Republic Of

## Abstract

**Introduction:**

There are animal models associating dopamine dysfunction with behavioral impairments that model attention deficit hyperactivity disorder (ADHD). Erythropoietin (EPO) has trophic effects on dopaminergic neurons.

**Objectives:**

The aim of this study was to examine the Erythropoietin (EPO) plasma levels and determine whether there was any correlation between plasma EPO levels and clinical characteristics of Attention-Deficit/Hyperactivity Disorder(ADHD).

**Methods:**

Plasma EPO levels were measured in 78 drug naïve children with ADHD and in 81 healthy children. The severity of ADHD symptoms was determined by scores on the Korean ADHD Rating Scale (K-ARS) in children and healthy controls.

**Results:**

The ADHD group consisted of 64 boys and 14 girls, and the healthy control group of 31 boys and 50 girls. The median plasma EPO levels in ADHD children was 12.9 mIU/mL, whereas it was 12.0 mIU/mL in the healthy controls. This difference was not statistically significant. Participants in the highest tertiles of plasma EPO had a 1.49 times higher risk of ADHD than those in the lowest tertile, and those in the second highest tertile had a 2.39 times higher risk of ADHD than those in the lowest tertile. Plasma EPO levels correlated positively with some K-ARS scores, including hyperactivity-impulsivity score and total score. The significant difference in hyperactivity-impulsivity score comparing participants in the second highest with those in the lowest tertile. total K-ARS score was significantly higher in the second highest tertile of plasma EPO compared to those in the lowest tertile.

**Conclusions:**

These findings suggest that plasma EPO levels were related to some ADHD symptoms, which could be used in the monitoring of the disorder. Further studies are required to clearly understand the source and role of EPO in ADHD.

**Disclosure of Interest:**

None Declared

